# Dioscin Improves Pyroptosis in LPS-Induced Mice Mastitis by Activating AMPK/Nrf2 and Inhibiting the NF-*κ*B Signaling Pathway

**DOI:** 10.1155/2020/8845521

**Published:** 2020-12-31

**Authors:** Xin Ran, Yan Zhang, Yuanxi Yang, Guiqiu Hu, Juxiong Liu, Shuang Hou, Wenjin Guo, Xingchi Kan, Shoupeng Fu

**Affiliations:** ^1^College of Animal Science and Veterinary Medicine, Jilin University, Changchun 130062, China; ^2^Hospital of Stomatology, Jilin University, Changchun 130021, China

## Abstract

Dioscin, a natural steroid saponin, has been shown to have anti-inflammatory effects, but its protective mechanism against mastitis is still unknown. NLRP3 inflammasome and pyroptosis play important roles in the pathogenesis of many inflammatory diseases, including mastitis. The purpose of this study was to explore the effect of dioscin on lipopolysaccharide- (LPS-) induced mastitis in vivo and in vitro and its mechanism of action. In vivo experiments, dioscin can reduce the inflammatory lesions and neutrophil motility in mammary tissue. Moreover, dioscin also can reduce the production of proinflammatory factors such as interleukin-1 beta (IL-1*β*) and inhibit the activation of NLRP3 inflammasome in LPS-induced mice mastitis. In vitro experiments, the results showed that dioscin inhibited the inflammatory response and the activation of NLRP3 inflammasome, but the survival rate of mouse mammary epithelial cells (mMECs) induced by LPS+ATP is increased. Subsequently, the experiment convinces that dioscin can reduce LPS+ATP-induced mMEC pyroptosis by adding Ac-DEVD-CHO (a caspase-3 inhibitor). Further mechanistic studies demonstrate that dioscin can activate AMPK/Nrf2 to inhibit NLRP3/GSDMD-induced mMEC pyroptosis. In summary, this paper reveals a novel function of dioscin on mMEC pyroptosis and provides a new potential therapy of dioscin for the treatment and prevention of mastitis.

## 1. Introduction

Cow mastitis is a significant disease that affects the dairy products in production and quality [1]. A lot of factors can cause mastitis, especially the pathogen infections and what we usually found is Gram-negative bacteria causes the mastitis in cow [2, 3]. Therefore, lipopolysaccharide (LPS), as the main pathogenic component of Gram-negative bacteria, has been used in a variety of mastitis animal models [4]. Previous studies have shown that mammary epithelial cells have immune activity, which can activate NOD-like receptor protein 3 (NLRP3) inflammasome [5] and nuclear factor kappa B (NF-*κ*B) signaling pathways [6] during mastitis, and that can release proinflammatory cytokines such as interleukin-1 (*IL-1β*), interleukin-6 (*IL-6*), and tumor necrosis factor alpha (*TNF-α*). Thereby, the effects of expanding the inflammatory response and promoting the exacerbation of inflammation become obvious [7, 8]. Recent studies have shown that pyroptosis induced by the activation of NLRP3 inflammasome is a significant cause of proinflammatory cytokine release [9, 10]. Therefore, inflammasomes and pyroptosis may play key roles in the inflammatory process of mastitis.

Pyroptosis has long been mistaken for a type of apoptosis caused by an identified pathogen [11]. However, recent studies have demonstrated that pyroptosis as a form of inflammatory regulatory cell death is distinct from apoptosis [12, 13]. There is an increasing evidence that inflammasomes can induce pyroptosis in a variety of cells, and because of this, many infectious diseases will be caused. This process plays an important role in a wide range of infectious diseases [14, 15]. Inflammasomes, including NLRP3, are a group of protein complexes and also act as pathogen recognition receptors to recognize danger signals and promote inflammation [16, 17]. NLRP3, the most characteristic inflammasomes, usually is activated by a variety of “danger signals,” such as ATP and reactive oxygen species (ROS) that recruit ASC and promote caspase-1 activation. The activated caspase-1 cleaves Gasdermin D (GSDMD) protein to the N- and C-terminals and leads to enzymatic hydrolysis of inflammatory factors, for instance, IL-1*β*. Then, the N-terminal fragment of GSDMD results in membrane perforation and the release of proinflammatory cytokines and lactate dehydrogenase (LDH) [10, 18–20]. However, whether the activation of NLRP3 inflammasome in mammary epithelial cells induces pyroptosis has not been reported.

At present, antibiotics are generally used in the treatment of cow mastitis, and the former practices of combining antibiotics with anti-inflammatory drugs are good examples to heal with the mastitis. Therefore, the selection of anti-inflammatory drugs still is a main therapy in curation of mastitis [21–23]. Dioscin, extracted from the tubers of Dioscorea japonica, is a natural compound. As for pharmacological effects, such as antioxidant, anti-inflammatory and antiapoptosis are also its characteristics [24–28]. Previous studies provide many documents in which dioscin, activated nuclear factor erythroid 2-related factor 2 (Nrf2), has an anti-inflammatory effect and is an inhibitor of NLRP3 inflammasome [25, 29]. However, whether dioscin prevents or mitigates the pathogenesis of mastitis through inhibiting pyroptosis caused by inflammasomes of mammary epithelial cells is still unknown. Therefore, in this study, the protective role and mechanism of dioscin in mice model of mastitis induced by LPS are investigated.

## 2. Material and Methods

### 2.1. Animal

BABL-/-c (8/9 weeks old) mice were purchased from the Experimental Animal Center of Bethune Medical College of Jilin University and were distributed in a small cage with two males and two females at 22°C-23°C, 12 h light and dark cycle, and ad libitum access to food and water. When the female mouse was pregnant, it was transferred to a separate small cage. This study is approved by the development of Animal Care and Use Committee of Jilin University, and all animal care and experimental procedures are under the supervision of this committee (approved on 27 February 2015, Protocol No. 2015047).

### 2.2. Mastitis Model

The female mice who were delivering after 5-7 days were randomly assigned to 5 groups. The groups are as follows: control group, dioscin group, LPS-induced mastitis group, and LPS-induced mastitis groups treated with dioscin or dexamethasone. Except for the NT group and the dioscin group, the mouse model of mastitis was induced by LPS in other groups. Dioscin (45 mg/kg/day) was administered orally, and dexamethasone (5 mg/kg/day) was injected intraperitoneally, starting from 3 days before LPS-induced mastitis. Before the mastitis was induced for 3 h, the female mice with their pups were separated. Mice were anesthetized by pentobarbital, and their fourth pair of nipples was disinfected with 75% alcohol. The 1 mm of the nipples was cut off with sterilizing scissors. Then, the mice were injected with 10 *μ*g LPS (0.2 mg/ml) into their nipples with 32G needle. Mastitis was induced 24 h later, and all mice were sacrificed, and samples of mammary tissues were taken to detect.

### 2.3. Cell Culture and Treatment

mMECs were purchased from the American Type Culture Collection (ATCC, ATCC® CRL-3063™), cultured in DMEM (Gibco, Grand Island, NY 14072, USA) with 10% FBS (Clark Bioscience, Richmond, VA, USA), and maintained at 37°C in a humidified chamber of 5% CO_2_. The mMECs' density reached 80%-90% in the cell culture flask. The cells were seeded into 60 mm × 15 mm cell culture dishes, 6-well plates, or 96-well plates. When the mMECs' density reached 70%, they were preprotected with dioscin for 2 h, then stimulated with LPS (1 *μ*g/ml) for different time (1 h, 6 h, and 24 h), 30 min or 1 h before LPS stimulation was completed, plus ATP (5 mM) stimulation. After stimulation, cell samples were collected for subsequent experiments.

### 2.4. Histopathological examination

The mammary tissues were fixed in 4% formaldehyde for 24 hours, then placed in different concentrations of alcohol and xylene in turn, fixed with paraffin, and cut into 5-micron thick sections. They were stained with eosin and hematoxylin and we observed histopathological changes under a light microscope. Three skilled pathological section analysts were invited to score the paraffin sections. Five fields of view were selected for each section to observe the degree of neutrophil aggregation, acinar integrity, and changes in acinar wall thickness. The scoring criteria are varying from 0 points to 4 points in no injury, mild injury, moderate injury, severe injury, and extreme injury, respectively.

### 2.5. Immunohistochemistry

For immunohistochemical detection of GSDMD on paraffin sections of mammary tissue, 5 *μ*m thick sections of mammary tissue fixed with paraffin were cut. Dewaxed and hydrated, the sections were digested with PBS containing 0.01% trypsin (Sigma) for 5 minutes, and then, the sections were heated in a 10 mmol/L citrate buffer (pH 6.0) to 90°C and stopped heating and cooled to 70°C, repeated 2-3 times. And cooling the section waits for down to the normal temperature. After treatment, sections were treated with 3% catalase to block endogenous peroxidase activity and subsequently blocked with 0.5% blocking agent for 30 minutes to reduce the number of nonspecific reactions. Sections were incubated with antibodies against GSDMD (diluted 1 : 500 in buffer) (Santa Cruz, USA) at 4°C overnight. Subsequently, wash 3 times with PBS for 5 minutes, and a biotinylated linking antibody solution (Biozol, Eching, Germany) was applied (100 *μ*L/section) for 20 min at 37°C. Sections should be kept in incubation with antibodies against GSDMD (diluted 1 : 500 in buffer) (Santa Cruz, USA) at 4°C for one night. After washing with PBS again, slides were stained with DAB (IBL, Germany) counterstained with hematoxylin. The negative control used affinity-purified preimmune IgG instead of the primary antibody.

### 2.6. Myeloperoxidase (MPO)

Taking a part of mammary tissue sample and adding hepesfreeacid (HEPES) according to the proportion are the first procedures. After fully grinding the sample, the supernatant should be better for enzyme-linked immunosorbent assay (ELISA). Later, cetyltrimethylammonium chloride (CTAC) to the precipitate in proportion is added to the sample, and the supernatant is used for myeloperoxidase (MPO) after sufficient grinding. Then, detect the absorbance peak at 450 nm. And the concentration of the analyte is determined based on the OD value.

### 2.7. ELISA

After mammary tissue is added to HEPES in proportion, the tissue is grinding and the supernatant is taken. Then, the tissue homogenate was used to gauge the concentrations of IL-1*β*, IL-6, and TNF-*α* under the instruction of the manufacturer (Biolegend, San Diego, CA, USA).

### 2.8. Western Blot Analysis

The SN of mastitis mice and mMECs were obtained for Western blot assay, and the experimental procedures are as shown in the previous experiments [30]. The membranes were incubated with primary antibodies against INOS (1 : 2000), COX-2 (1 : 1000), caspase-3 (1 : 1000), Nrf2 (1 : 2000), phosphor-NF-*κ*B p65 (1 : 1000). NF-*κ*Bp65 (1 : 1000), p-AMPK (1 : 2000), AMPK (1 : 2000), lamin B (Cell Signaling Technology, Danvers, MA, USA), and NLRP3 (1 : 2000), ASC (1 : 2000), caspase-1 (1 : 2000), GSDMD (1 : 1000), and *β*-actin (1 : 2000) (Santa Cruz Biotechnology Inc., Santa Cruz, CA, USA) at 4°C overnight. Subsequently, the membranes were incubated with secondary goat anti-rabbit or anti-mouse or anti-goat (1 : 5000; Santa Cruz, CA, USA) antibody for 1 hour at room temperature. Membranes were visualized after using enhanced chemiluminescence (ECL kit; Applygen Inst. Biotech, Beijing, China).

### 2.9. Quantitative Real-Time PCR

Total RNA was extracted from mouse mammary tissue and mMECs by using Trizol (Invitrogen, Carlsbad, CA, USA), and RNA transcription was performed adopting the PrimeScript^TM^ RT Reagent Kit (TaKaRa, Japan). Quantitative real-time RT-PCR was performed through using a SYBR Green PCR Master Mix (Roche, South San Francisco, CA, USA). The mRNA expression of TNF-*α*, IL-6, IL-1*β*, GSDMD, caspase-1, caspase-3, ASC, and NLRP3 was normalized to the mRNA expression of *β*-actin. The primer sequences are demonstrated in Table 1.

### 2.10. CCK-8

CCK-8 was added to determine the effect of dioscin on the activity of mMECs. Six different concentrations of dioscin were selected (25, 50, 100, 200, 400, 800, 1600, and 2400 *μ*g/ml). After adding dioscin of different concentrations in mMEC culture medium for 24 hours, 10 *μ*L (Saint-Bio, Shanghai, China) CCK-8 was added. After 1 hour, the OD value at 450 nm was measured by a microplate reader.

### 2.11. Cell Death Analysis

The treated mMECs were washed three times with PBS stored at 4°C. Apoptosis was detected by using the TUNEL apoptosis detection kit (KeyGEN BioTECH, Nanjing, China). Tunel-positive cells had stained red nuclei, and DAPI-stained cells had blue nuclei. We chose the Annexin V-FITC/PI apoptosis detection kit (Beyotime Institute of Biotechnology, China) to further detect the mid-stage and late-stage apoptosis of mMECs according to the instruction manual. Apoptosis index expressed as a ratio of the whole number of positive apoptotic cells.

### 2.12. Immunofluorescence Assay

After inoculating mMECs to a 24-well plate for 24 h, pretreated with dioscin (400 *μ*g/ml) for 1 h, LPS (1 *μ*g/ml) was stimulated for 1 h, and before LPS stimulation was completed, ATP (5 mM) was added for stimulation. Immunocytochemistry-immunofluorescence (ICC-IF) experiment was used to detect the nuclear translocation of Nrf2 and NF-*κ*B p65. Analysis of nuclear translocation of Nrf2 and NF-*κ*B p65 subunits was done through using anti-Nrf2 (1 : 500 Abcam, UK, Cambridge, USA) and anti-NF-*κ*B p65 antibody (1 : 1000 Cell Signaling Technology, USA) [31].

### 2.13. Cytotoxicity Assay

Cytotoxicity was determined by the release of lactate dehydrogenase (LDH). In this experiment, the LDH kit (Beyotime Institute of Biotechnology, China) was used for the test.

### 2.14. ROS Detection

The ROS kit (Beyotime Institute of Biotechnology, China) was used to detect the intracellular reactive oxygen content.

### 2.15. Statistical Analysis

The experimental data were analyzed by GraphPad Prism7 (Manufacturer, La Jolla, CA, USA). Differences between groups were analyzed by one-way analysis of variance (ANOVA), and pairwise comparisons were performed by LSD. *p* values of less than 0.05 were considered statistically significant (^#^significant compared with the NT group; ^∗^*p* < 0.05; ^∗∗^*p* < 0.01).

## 3. Results

### 3.1. Dioscin Ameliorates LPS-Induced Mastitis in Mice

**Figure 1:**
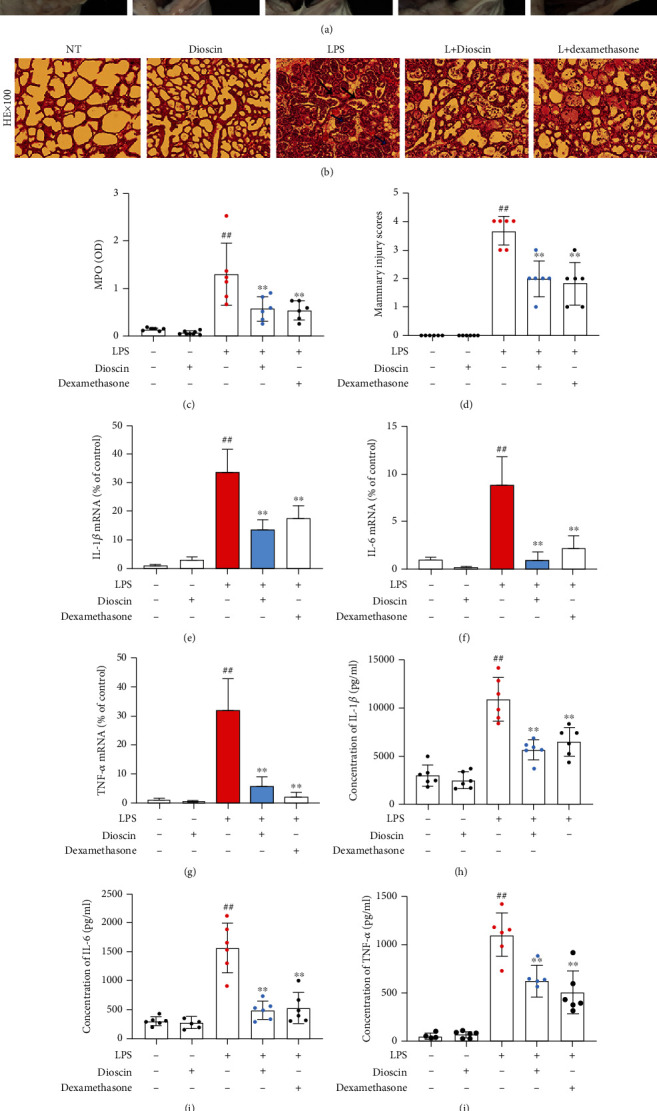
Dioscin ameliorates LPS-induced mastitis in mice. (a) Photographs of mammary gland tissue from mice in different treatment groups. (b) HE staining of mammary gland tissue in mice in different treatment groups. The blue arrow represents the accumulation of neutrophils in the acinar cavity, and the black arrow represents thickening of the acinar membrane. The scale bar represents 500 *μ*m. (c) Detection of MPO activity in mammary tissue. (d) Histopathological score of mammary tissue. The mRNA levels of (e) IL-1*β*, (f) IL-6, and (g) TNF-*α* in mammary tissue homogenate. The protein levels of (h) IL-1*β*, (i) IL-6, and (j) TNF-*α* in mammary tissue homogenate. ^#^*p* < 0.05 and ^##^*p* < 0.01 compared to the control group; ^∗^*p* < 0.05 and ^∗∗^*p* < 0.01 compared to the LPS group.

In order to investigate the protective effect of dioscin on mastitis mice, the following tests were experimented on the mammary tissue of mice in different treatment groups. The result showed that dioscin administration reduces swelling and congestion of mammary tissue induced by LPS (Figure 1(a)). HE staining showed that LPS stimulation caused hyperemic edema in the acinar cavity, and acinar cavity was infiltrated with a large amount of polymorphonuclear leukocytes; the addition of dioscin significantly reduced the histopathological changes induced by LPS (Figures 1(b) and 1(d)). The MPO test further proved that the addition of dioscin could significantly reduce the inflammatory cell infiltration induced by LPS (Figure 1(c)). In addition, the results also witnessed that LPS increased the expression and secretion of proinflammatory cytokines such as IL-1*β* (Figures 1(b) and 1(h)), IL-6 (Figures 1(f) and 1(i)), and TNF-*α* (Figures 1(g) and 1(j)), but the pretreated of dioscin significantly reduced them.

### 3.2. Dioscin Ameliorates Inflammation and Death of mMECs Induced by LPS+ATP

**Figure 2:**
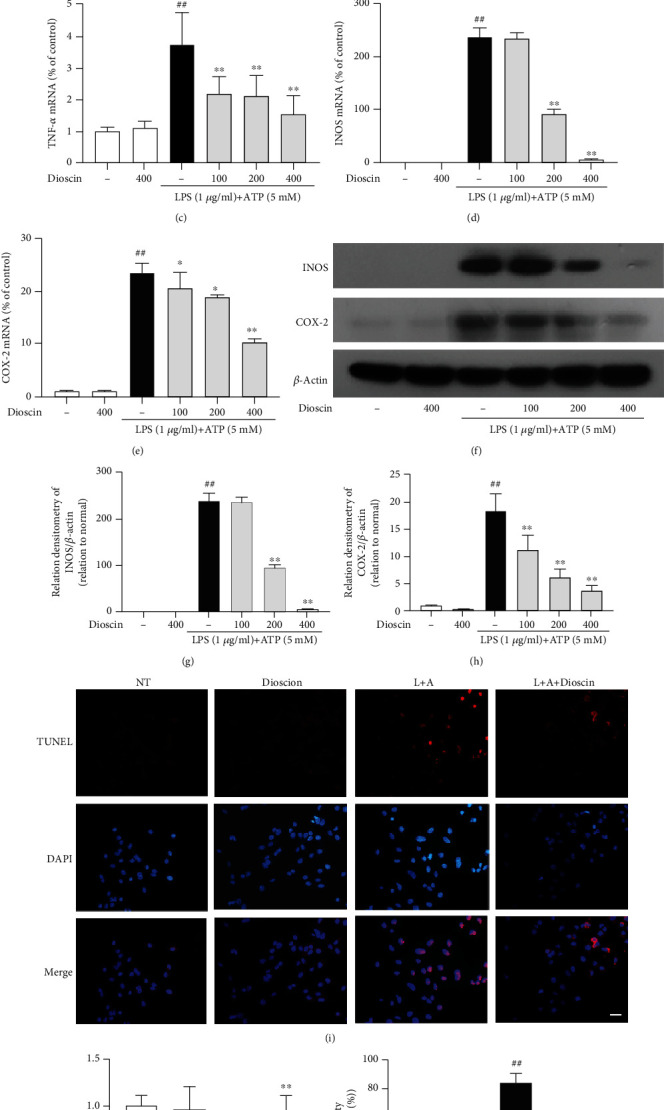
Dioscin ameliorates inflammation and death of mMECs induced by LPS+ATP. mMECs were pretreated with dioscin at different concentrations (100 ng/ml, 200 ng/ml, and 400 ng/ml) for 2 h, and LPS (1 *μ*g/ml) was stimulated for 12 h, and then, ATP (5 mM) was added for 30 min before the LPS stimulation was completed. (a–e) The mRNA levels of IL-1*β*, IL-6, TNF-*α*, INOS, and COX-2 were examined by RT-PCR. (f–h) The protein levels of INOS and COX-2 were examined by Western blot. Relative protein levels of them were normalized to *β*-actin. The mMECs were pretreated with dioscin (400 ng/ml) for 2 h, LPS was stimulated for 24 h, and then ATP (5 mM) was added 1.5 h before LPS stimulation was completed. (i) TUNEL staining of treated mMECs. The scale bar represents 200 *μ*m. The green arrow represents the positive TUNEL cells. (j) mMEC survival rate measured by CCK-8 kit. (k) The cell LDH release was detected by LDH kit. ^#^*p* < 0.05 and ^##^*p* < 0.01 compared to the control group; ^∗^*p* < 0.05 and ^∗∗^*p* < 0.01 compared to the LPS+ATP group.

To investigate the anti-inflammatory mechanism of dioscin, the experiments were designed to detect the effect of dioscin on LPS+ATP-stimulated mMECs. The study also has been found that less than 800 ng/ml of dioscin has no cytotoxicity on mMECs (supplementary Fig. S1). Stimulation of mMECs with LPS+ATP significantly increased gene expression of the proinflammatory mediators IL-1*β*, IL-6, TNF-*α*, INOS, and COX-2 (Figures 2(a)–2(e)) and protein expression of INOS and COX-2 (Figures 2(f) and 2(h)). As expected, dioscin significantly inhibited these responses in a dose-dependent manner. In addition, pretreatment with dioscin significantly reduced LPS+ATP-induced cell death (Figures 2(i) and 2(j)) and LDH release (Figure 2(k)). These results suggest that dioscin has a positive effect on cell viability of mMECs induced by LPS+ATP.

### 3.3. Dioscin Reduces LPS+ATP-Induced Activation of NF-*κ*B Signaling Pathway and NLRP3 Inflammasome in mMECs

**Figure 3:**
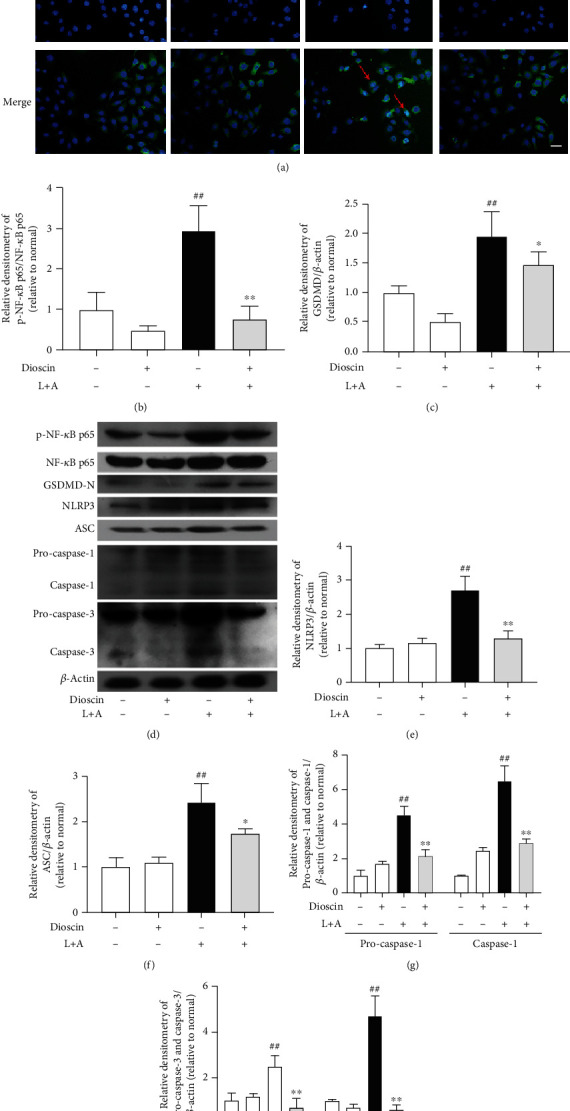
Dioscin reduces LPS+ATP-induced activation of NF-*κ*B signaling pathway and NLRP3 inflammasome in mMECs. The mMECs were pretreated with dioscin (400 ng/ml) for 2 h before stimulated with LPS for 1 h, and ATP (5 mM) was added 30 minutes before the end of stimulation. (a) Nuclear translocation of NF-*κ*B p65 subunit measured by immunofluorescence. The scale bar represents 200 *μ*m. The red arrow represents the nuclear translocation of NF-*κ*B p65. (b) The phosphorylation of p65 was detected using Western blot. The phosphorylation ratio of NF-*κ*B p65 was quantified. mMECs were pretreated with dioscin for 2 h, then stimulated with LPS for 6 hours, and added ATP (5 mM) 30 min before LPS stimulation was completed. (c, d) GSDMD-N, (e) NLRP3, (f) ASC, (g) caspase-1, and (h) caspase-3 protein expressions were measured using Western blot, expressed as densitometry quantitation using *β*-actin as an internal control. ^#^*p* < 0.05 and ^##^*p* < 0.01 compared to the control group; ^∗^*p* < 0.05 and ^∗∗^*p* < 0.01 compared to the LPS+ATP group.

NF-*κ*B signaling pathway and NLRP3 inflammasome play an important role in regulating the production of proinflammatory cytokines in the course of inflammation. The NF-*κ*B signaling pathway and NLRP3 inflammasome in LPS+ATP-induced mMECs were investigated. The results showed that dioscin significantly ameliorated the phosphorylation of NF-*κ*B p65 induced by LPS+ATP in mMECs (Figure 3(b)). Previous studies have shown that the activation of NF-*κ*B can also lead to nuclear translocation of NF-*κ*B p65 subunit [32]. The experimental results from the studies of this paper did show that LPS+ATP induced nuclear translocation of NF-*κ*B p65, and pretreatment of dioscin significantly inhibited nuclear translocation of NF-*κ*B p65 (Figure 3(a)). Detection of inflammasome found that LPS+ATP significantly increased the expression of NLRP3, ASC, and caspase-1 of mMECs at the protein level (Figures 3(e)–3(g)), and NLRP3 inflammasome gene level test results were consistent with protein levels (Supplementary Fig. S2a-d). To our surprised, pretreatment dioscin also reduced the protein levels of cleaved caspase-1 and cleaved caspase-3 (Figures 3(g) and 3(h)). These results indicated that dioscin not only inhibited the expression of NLRP3 inflammasome but also hindered its activation. Besides, LPS caused an increase in GSDMD-N, a key protein of pyroptosis, in LPS+ATP-induced mMECs at protein and gene levels, and dioscin significantly improved this phenomenon (Figure 3(c), Supplementary Fig. S2e).

### 3.4. Dioscin Reduces Pyroptosis of mMECs Induced by LPS+ATP

**Figure 4:**
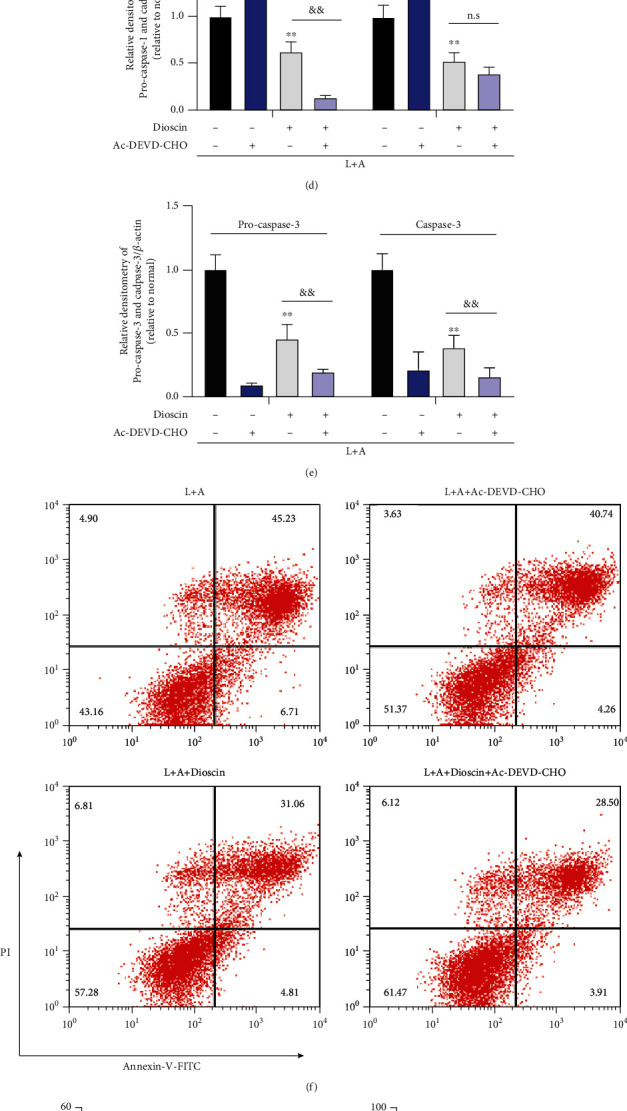
Dioscin reduces pyroptosis of mMECs induced by LPS+ATP. mMECs were treated with Ac-DEVD-CHO (20 *μ*M) for 2 h, followed by dioscin (400 ng/ml) for 2 h, LPS (1 *μ*g/ml) stimulation for 6 h, and ATP (5 mM) was added 30 minutes before the end of stimulation. The mRNA levels of (a) caspase-1, (b) caspase-3, and (j) IL-1*β* were examined by RT-PCR. (c) The protein levels of (d) caspase-1 and (e) caspase-3 were measured by Western blot and quantified by comparison with *β*-actin. Cells were treated with Ac-DEVD-CHO (20 *μ*M) for 2 h, followed by dioscin (400 ng/ml) for 2 h, and LPS (1 *μ*g/ml) stimulation for 24 h, and ATP (5 mM) was added 1.5 h before the end of stimulation. (f) The cell death was detected by flow cytometry and analysis of pyroptosis cells. (g) The Annexin^+^ PI ^+^ cells in flow cytometry were quantified. (h) The cell LDH release was detected by LDH kit. (i) The production of IL-1*β* was detected by ELISA. ^∗^*p* < 0.05 and ^∗∗^*p* < 0.01 compared to the LPS+ATP group; ^&^*p* < 0.05 and ^&&^*p* < 0.01 compared to the dioscin group.

To investigate the unique effect of dioscin on apoptosis or pyroptosis, Ac-DEVD-CHO was used, a specific caspase-3 inhibitor, to inhibit caspase-3-induced apoptosis. The results showed that after adding Ac-DEVD-CHO, the activation of caspase-3 stimulated by LPS+ATP was significantly inhibited (Figures 4(b), 4(c), and 4(e)), but caspase-1 had no effect (Figures 4(a), 4(c), and 4(d)). Dioscin still significantly reduced the activation of caspase-1 induced by LPS+ATP after using Ac-DEVD-CHO (Figures 4(c) and 4(d)). Flow cytometry was chosen to observe how dioscin affected cell death. Interestingly, the results were consistent with caspase-1. Dioscin significantly reduced LPS+ATP-induced cell death, while caspase-3 had no effect on cell death (Figures 4(f) and 4(g)). These results led to speculate that dioscin ameliorated pyroptosis, not apoptosis. IL-1 secretion and LDH release are important indicators of pyroptosis, and the results showed that the inhibition of IL-1 secretion and LDH release by dioscin has no dependence on caspase-3 (Figures 4(h)–4(j)). Therefore, the hypothesis is that dioscin can reduce the mMEC pyroptosis induced by LPS+ATP.

### 3.5. Dioscin Activates AMPK-Nrf2 in LPS+ATP-Induced mMECs

**Figure 5:**
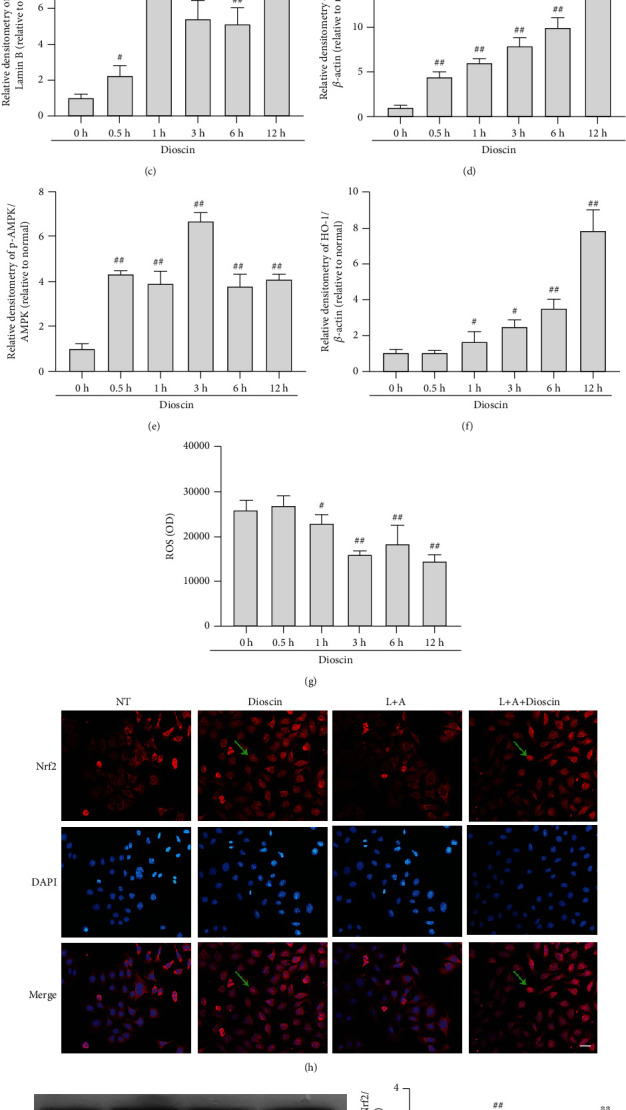
Dioscin activates AMPK-Nrf2 in LPS+ATP-induced mMECs. Dioscin (400 ng/ml) was pretreated for mMECs for different times (0, 0.5 h, 1 h, 3 h, 6 h, and 12 h). (a) The protein expressions of HO-1, p-AMPK, and AMPK were determined by Western blot. (b) Cytoplasmic, (c) nuclear, and (d) total levels of Nrf2 were determined by Western blot. (e) The phosphorylation ratio of AMPK was quantified. (f) HO-1 was quantified by comparison with *β*-actin. (g) The ROS content was determined by ROS detection kits. The mMECs were pretreated with dioscin (400 ng/ml) for 2 h, and LPS (1 *μ*g/ml) was stimulated for 6 h, and 30 min before LPS stimulation was completed, ATP (5 mM) was added for stimulation. (h) Nuclear translocation of Nrf2 protein was detected by immunofluorescence; the scale bar represents 200 *μ*m. The green arrow represents the nuclear translocation of Nrf2 protein. (i) The protein of nuclear (j) Nrf2 and (k) HO-1 was detected by Western blot. ^#^*p* < 0.05 and ^##^*p* < 0.01 compared to the control group; ^∗^*p* < 0.05 and ^∗∗^*p* < 0.01 compared to the LPS+ATP group.

Previous studies have shown that activating Nrf2 is a significant factor in the activation of NLRP3 inflammasome and phosphorylation of NF-*κ*B. Dioscin has been reported to be an antioxidant [25, 33], so the Nrf2 signaling pathway is the main work to do. The dioscin treated mMECs for different time periods (0, 0.5, 1, 3, 6, and 12 h), and the results showed that dioscin increased the protein expression of total Nrf2 and nuclear Nrf2 and phosphorylation of AMPK and HO-1 (Figures 5(a) and 5(c)–5(f)), decreased the protein of Nrf2 in the cytoplasm, and reduced reactive oxygen (Figures 5(a), 5(b), and 5(g)). Previous studies suggested that AMPK is an upstream of Nrf2 [34]. The results showed dioscin also promoted AMPK phosphorylation. Then compound C and ML385 (AMPK phosphorylation inhibitor and Nrf2 inhibitor) were added in the experiments, and what was found was that dioscin can reduce ROS production by increasing AMPK phosphorylation to increase Nrf2 activation (Supplementary Fig. 3s). Further research showed that dioscin could provoke Nrf2 and HO-1 to express after LPS+ATP stimulated mMECs (Figures 5(i)–5(k)). As predicted, dioscin enhanced nuclear translocation of the Nrf2 protein and caused significant activation of Nrf2 (Figure 5(h)).

### 3.6. Dioscin Reduces LPS+ATP-Induced Pyroptosis of mMECs by Activating Nrf2

**Figure 6:**
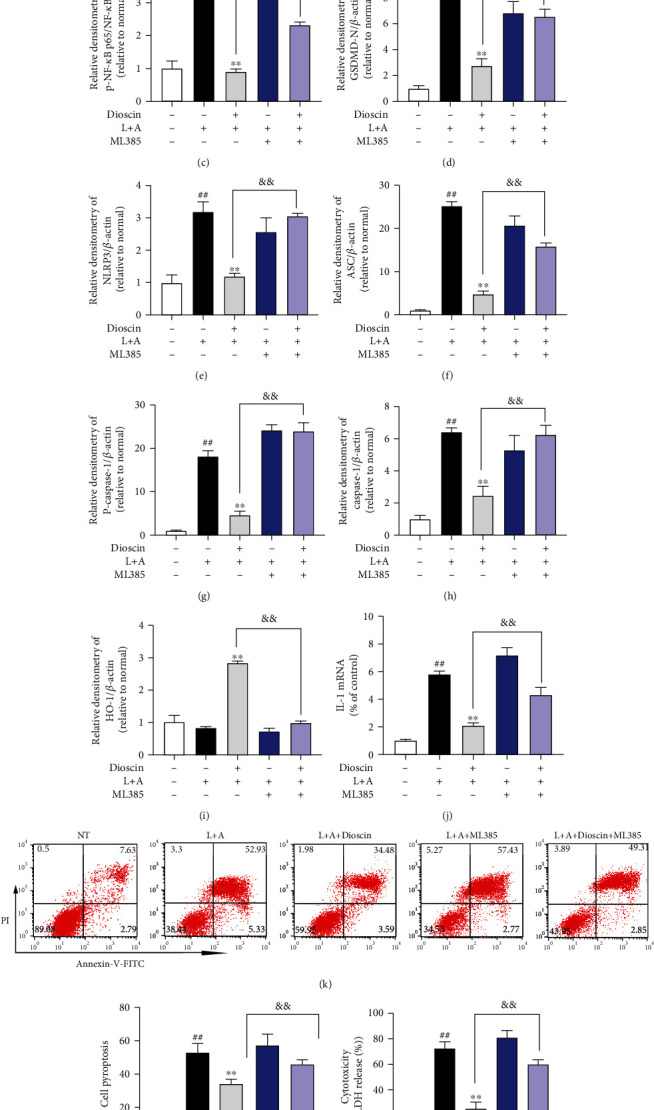
Dioscin reduces LPS+ATP-induced pyroptosis of mMECs by activating Nrf2. mMECs were treated with ML385 (5 *μ*M) for 4 hours before adding dioscin (400 ng/ml) for 2 hours, followed by LPS (1 *μ*g/ml) stimulation for 6 hours, and ATP (5 mM) was added 30 minutes before the end of stimulation. (a–j) The protein of nuclear Nrf2, p65, pp65, GSDMD-N, NLRP3, ASC, pro-caspase-1, caspase-1, and HO-1 was detected by Western blot. mMECs were treated with ML385 (5 *μ*M) for 4 h before adding dioscin (400 ng/ml) for 2 h, followed by LPS (1 *μ*g/ml) stimulation for 24 h, and ATP (5 mM) 1.5 h before the end of stimulation. (k, l) The cell pyroptosis was detected by flow cytometry. (m) Different treatment groups of mMECs were detected, and LDH release was detected by LDH kit. (n) The production of IL-1*β* was detected by ELISA. ^#^*p* < 0.05 and ^##^*p* < 0.01 compared to the control group; ^∗^*p* < 0.05 and ^∗∗^*p* < 0.01 compared to the LPS+ATP group; ^&^*p* < 0.05 and ^&&^*p* < 0.01 compared to the dioscin+LPS+ATP group.

We added the specific inhibitor of Nrf2, named ML385, to further determine whether the anti-inflammatory and antipyroptosis effects of dioscin were activated by Nrf2 (Figures 6(a), 6(b), and 6(i)). ML385 treatment significantly reduced the inhibitory effect of dioscin on NF-*κ*B p65 phosphorylation (Figures 6(a) and 6(c)). ML385 significantly relieved dioscin from inhibiting the activation of NLRP3 inflammasome (including NLRP3, ASC, and caspase-1) (Figures 6(a) and 6(e)–6(h)).

To our surprise, we subsequently found that ML385 significantly reduced the inhibitory effect of dioscin on GSDMD-N (Figures 6(a) and 6(d)). LDH and flow cytometry experiments showed that ML385 also significantly reduced the improvement effect of dioscin on pyroptosis of mMECs (Figures 6(k) and 6(m)). The secretion of IL-1*β* is consistent with the above results (Figure 6(n)). In summary, diocin reduces LPS-induced pyroptosis of mMECs by activating Nrf2.

### 3.7. Dioscin Inhibits Activation of NF-*κ*B Signaling Pathway and NLRP3 Inflammasome in LPS-Induced Mice Mastitis

**Figure 7:**
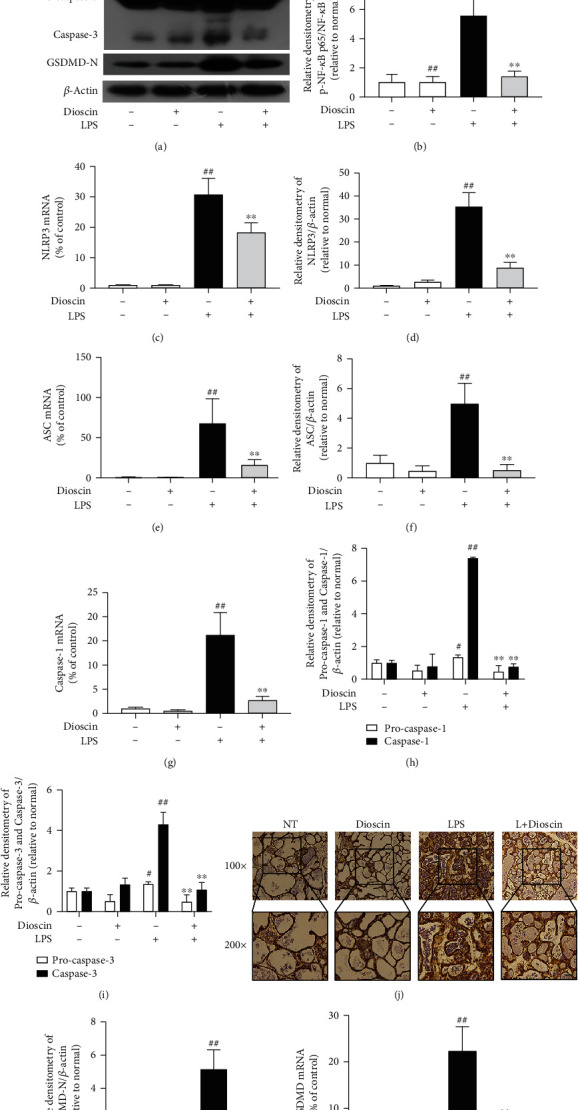
Dioscin inhibits activation of NF-*κ*B signaling pathway and NLRP3 inflammasome in LPS-induced mice mastitis. The mRNA levels of (c) NLRP3, (e) ASC, (g) caspase-1, and (l) GSDMD were detected in mammary tissue homogenate. (a) Western blot analysis of (b) pp65, p65, (d) NLRP3, (f) ASC, (h) caspase-1, (i) caspase-3, and (k) GSDMD-N in mammary tissue homogenate. Relative protein levels of NLRP3, ASC, caspase-1, caspase-3, and GSDMD were normalized to *β*-actin. (j) Immunohistochemical staining for GSDMD; the scale bar represents 250 *μ*m and 500 *μ*m. ^#^*p* < 0.05 and ^##^*p* < 0.01 compared to the control group; ^∗^*p* < 0.05 and ^∗∗^*p* < 0.01 compared to the LPS+ATP group.

In vivo, we further confirmed the inhibitory effect of dioscin on NF-*κ*B signaling pathway and NLRP3 inflammasome. The result showed that feeding dioscin significantly reduced LPS-induced NF-*κ*B p65 phosphorylation in mastitis mice (Figures 7(a) and 7(b)). Subsequently, we tested the NLRP3 inflammasome in mice mammary tissue and found that NLRP3, ASC, and caspase-1 were significantly increased at the gene level and protein level under LPS stimulation (Figures 7(a) and 7(b)–7(h)). The trend is the same as in vitro results; dioscin markedly reduced NLRP3 inflammasome expression and production. As predicted, dioscin treatment also reduced the protein levels of cleaved caspase-1 and cleaved caspase-3 (Figures 7(a), 7(h), and 7(i)). Dioscin also inhibited LPS-induced GSDMD expression of gene and GSDMD-N protein in LPS-induced mice (Figures 7(k) and 7(l)). Immunohistochemistry further confirmed our results of GSDMD in mammary tissue of mice (Figure 7(j)).

### 3.8. Dioscin promotes the activation of Nrf2 in LPS-induced Mastitis Mice

**Figure 8:**
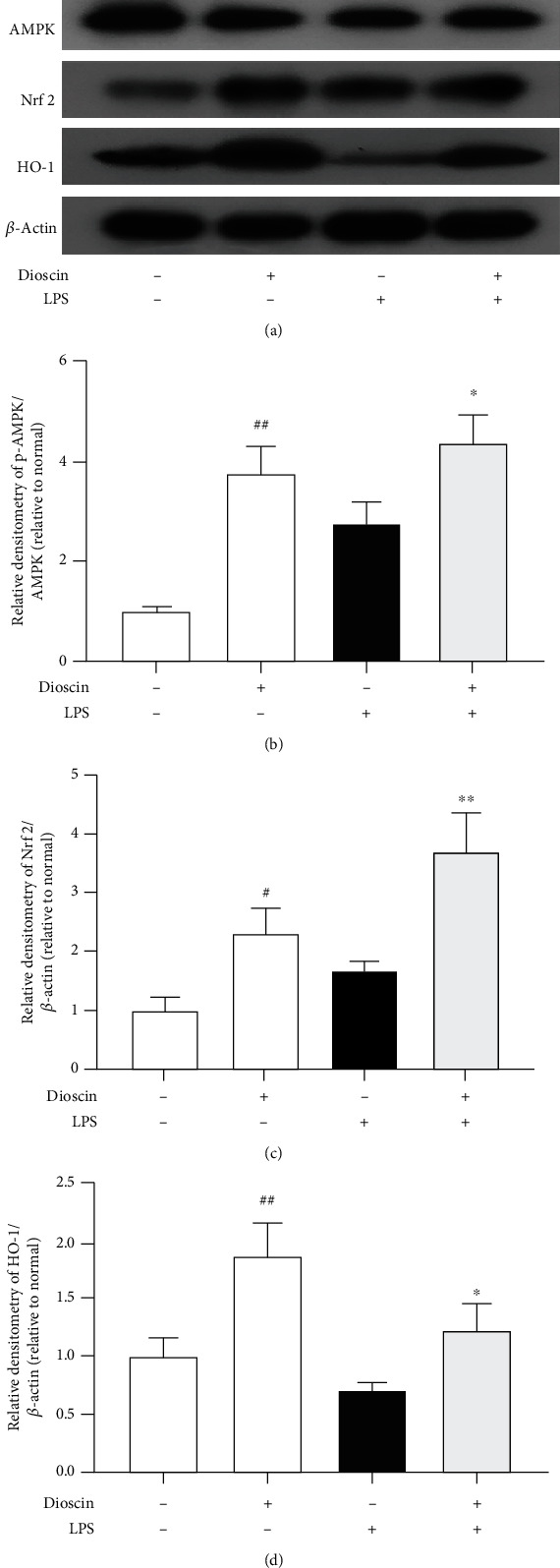
Dioscin promotes the activation of Nrf2 in LPS-induced mastitis mice. (a) Western blot analysis of AMPK, p-AMPK, and HO-1 in mammary tissue homogenate. (b–d) The quantitative analysis of Western blot results. ^#^*p* < 0.05 and ^##^*p* < 0.01 compared to the control group; ^∗^*p* < 0.05 and ^∗∗^*p* < 0.01 compared to the LPS group.

In vitro, dioscin has been shown to activate AMPK and Nrf2. For further expounding the mechanism of dioscin, the effect of dioscin on Nrf2 was also detected in LPS-induced mastitis mice. As was our anticipation, phosphorylation of AMPK and protein expressions of HO-1 and Nrf2 increased in mammary tissues of mice after intragastric administration with dioscin (Figure 8).

## 4. Discussion

Cow mastitis is a disease that will cause a great damage to dairy industry, and the therapy always relies on antibiotics mostly. However, the use of antibiotics is a promoter to raise the drug resistance of mastitis. Because of the drug resistance and high cost of antibiotics, the researchers are trying to select an appropriate combination of antibiotics and anti-inflammatory drugs to cure mastitis with a low cost but more effective. Therefore, that is why dioscin is into the eyes of researcher. This natural compound, dioscin, through the experiments, has many pharmacological effects including anti-inflammatory and also has virtues of high efficiency and low price [35]. To detect the therapeutic effect and mechanism of dioscin on mastitis, this experiment was designed in vivo and in vitro. In summary, in vivo, the classic model of LPS-induced mouse mastitis was introduced to the experiments [36]. In vitro, the application of LPS and ATP is to stimulate mMECs and to construct an in vitro mastitis model. Based on existent reports, two main interdependent processes will aggravate the mastitis inflammation: one is recruitment of inflammatory cells and the other is upregulation of proinflammatory cytokines [37]. In vivo, these experiments demonstrated that the dioscin reduces the mastitis symptoms induced by LPS in mice. HE staining and MPO of mouse mammary tissue intended that dioscin significantly ameliorates the pathological and histological changes of mammary and neutrophil infiltration caused by LPS. Previous studies have proved that reducing the production of proinflammatory cytokines, such as IL-1*β*, IL-6, and TNF-*α*, can reduce the effect of mastitis [38, 39]. Consistent with these studies, the dioscin obviously inhibited the production of proinflammatory cytokines IL-1*β*, IL-6, and TNF-*α* in vivo and in vitro.

After confirming the anti-inflammatory effect of dioscin in mastitis mice, the anti-inflammatory mechanism of dioscin was explored in the next step. During the occurrence of various inflammations, the NF-*κ*B pathway and NLRP3 inflammasome affect the subsequent development of inflammation through the regulation of inflammatory factors [40, 41]. The former way, NF-*κ*B pathway, relies on the regulation of expression on proinflammatory cytokines. To illustrate intensively, NF-*κ*B, a transcription factor mainly composed of p50/p65 heterodimer, regulates the expression of proinflammatory cytokines [42]. Activation of NF-*κ*B by LPS leads to the phosphorylation and nuclear translocation of NF-*κ*B p65, which not only increases the expression of trigger cytokine precursors, but also as an important initial step in the activation of NLRP3 inflammasome [43, 44]. But the latter, different with the NF-*κ*B pathway, NLRP3 inflammasome promotes maturation and secretion of cytokine. The expression of NLRP3, ASC, and caspas-1 increased significantly under the stimulation of LPS. Then, NLRP3 inflammasome is activated under the stimulation of damage-associated molecular patterns (DAMPs), such as ROS and ATP; then, ASC is recruited, caspase-1 is activated, and pro-IL-1*β* is processed into mature form [45]. Two independent and distinct forms of signals together induce the production of proinflammatory cytokines, for instance, IL-1*β*. This experiment ascertains that dioscin simultaneously inhibits the activation of NF-*κ*B signaling pathway and NLRP3 inflammasome, which implies that dioscin may have a stronger effect than the drug that only has single function way in anti-inflammatory.

Besides the production of intracellular proinflammatory cytokines, in the process of inflammation, the release of proinflammatory cytokines is also the key to trigger the inflammation and pyroptosis and is the main way to release the proinflammatory cytokine IL-1*β* from the cell [46]. Previous studies have certified that LPS stimulation leads to pyroptosis of multiple tissues and organs, such as intestinal epithelium, vascular endothelium, and adipose tissue [47–49]. However, whether LPS can induce pyroptosis in mMECs or the relationship between pyroptosis of mMECs and mastitis has not been reported. In this experiment, we demonstrated that dioscin can reduce LPS+ATP-induced mMEC death, but whether the reduction is due to pyroptosis is still unclear. It is worth noting that there have been studies justifying that dioscin regulates apoptosis in various diseases [28, 50]. Therefore, after the detection of caspas-3, we found that dioscin could reduce the expression and activation of caspas-3. In this study, we used Ac-DEVD-CHO, a specific inhibitor of caspase-3, to exclude the negative effect of caspase-3-mediated apoptosis on mMECs. Subsequent detection of LDH and IL-1*β* proved that dioscin reduced LPS+ATP-induced mMEC pyroptosis. Studies have proven that GSDMD is an indispensable factor in the occurrence of cell pyroptosis [51]. The N-terminal GSDMD fragment mediates the formation of cell membrane pores and directly triggers the occurrence of cell pyroptosis [46]. We speculate whether GSDMD is the crucial to dioscin regulating pyroptosis. Our results indicate that dioscin reduces the cell pyroptosis of mMECs by inhibiting the production of GSDMD-N. It has been reported that the transcription of GSDMD can be regulated by NF-*κ*B [49], so the speculation is raised that the dioscin is likely to inhibit GSDMD by regulating the activation of the NF-*κ*B pathway. This conjecture needs further verification.

After exploring, one thing can be sure that dioscin has antioxidant effects that can scavenge the production of ROS by activating Nrf2 [25]. While continuing and uncontrolled inflammation is closely related to oxidative stress, LPS promotes the infiltration of inflammatory cells and proinflammatory mediators to evoke more production of ROS; in turn, excessive ROS inflicts more severe inflammation, exacerbating tissue destruction [52–54]. In this experiment, dioscin inhibited the production of ROS by activating AMPK-Nrf2 in mastitis mice and mMECs. Recent studies exemplified that Nrf2 activation can inhibit inflammation-mediated damage through its antioxidant effect [55]. However, the effects of Nrf2 on the NF-*κ*B signaling pathway, NLRP3 inflammasome, and cell pyroptosis remain controversial [56, 57]. By adding ML385, Nrf2 inhibitors, the effect of Nrf2 on the NF-*κ*B signaling pathway, NLRP3 inflammasome, and cell pyroptosis in LPS+ATP-stimulated mMECs was investigated. The results intended that dioscin had an inhibitory effect on NF-*κ*B and NLRP3 in a Nrf2-dependent manner on one hand. On the other hand, dioscin may have other inhibitory pathways besides inhibiting mMEC pyroptosis by activating Nrf2.

In conclusion, dioscin improves pyroptosis in LPS-induced mice mastitis by activating AMPK/Nrf2 and inhibiting the NF-*κ*B signaling pathway. We demonstrated that dioscin significantly alleviated LPS-induced mastitis in mice, and the underlying mechanisms of this protective effect include antioxidant and anti-inflammatory. In summary, the results of this study provide experimental basis for the application of dioscin in the treatment of mastitis and will lead the way for the therapy of mastitis caused by Gram-negative bacterial infection. The further study of dioscin should focus its inhabitation function in mastitis and other similar diseases.

## Figures and Tables

**Table 1:** Primers used for qRT-PCR.

Gene	Sequence
*β*-Actin	F: 5′-GTCAGGTCATCACTATCGGCAAT-3′
	R: 5′-AGAGGTCTTTACGGATGTCAACGT-3′

TNF-*α*	F: 5′-CCACGCTCTTCTGTCTACTG-3′
	R: 5′-CCACGCTCTTCTGTCTACTG-3′

IL-1*β*	F: 5′-TGTGATGTTCCCATTAGAC-3′
	R: 5′-AATACCACTTGTTGGCTTA-3′

IL-6	F: 5′-AGCCACTGCCTTCCCTAC-3′
	R: 5′-TTGCCATTGCACAACTCTT-3′

INOS	F: 5′-GAACTGTAGCACAGCACAGGAAAT-3′
	R: 5′-CGTACCGGATGAGCTGTGAAT-3′

COX-2	F: 5′-CGTACCGGATGAGCTGTGAAT-3′
	R: 5′-CCAGCACTTCACCCATCAGTT-3′

NLRP3	F: 5′-GAGCTGGACCTCAGTGACAATGC-3′
	R: 5′-ACCAATGCGAGATCCTGACAACAC-3′

ASC	F: 5′-CAGGCGAGCAGCAGCAAGAG-3′
	R: 5′-CAAGAGCGTCCAGGATGGCATC-3′

Caspase-1	F: 5′-ACAACCACTCGTACACGTCTTGC-3′
	R: 5′-CCAGATCCTCCAGCAGCAACTTC-3′

Caspase-3	F: 5′-CGACTGGCGTGTGCGAGATG-3′
	R: 5′-AGCAGCAGCAGCAGCAACAG-3′

GSDMD	F: 5′-AGTTCCGCTCTTGGTCGTG-3′
	R: 5′-CTGCCGCTTACCTCCTTGAT-3′

## Data Availability

The data used to support the findings of this study are available from the corresponding author upon request.

## Supplementary Materials

Supplementary Materials Supplementary Figure S1: the cell cytotoxicity of dioscin on mMECs. (a) Chemical structure of dioscin. (b) MMEC viability was determined by CCK-8 assay. ^#^*p* < 0.05 and ^##^*p* < 0.01 compared to the control group. Supplementary Figure S2: the gene expression of NLRP3 inflammasome and GSDMD. (a–c) The mRNA levels of NLRP3, ASC, and caspase-1. (d) The mRNA levels of caspase-3. (e) The mRNA levels of GSDMD. ^#^*p* < 0.05 and ^##^*p* < 0.01 compared to the control group. ^∗^*p* < 0.05 and ^∗∗^*p* < 0.01 compared to the LPS+ATP group.

## References

[1] Viguier C., Arora S., Gilmartin N., Welbeck K., O'Kennedy R. (2009). Mastitis detection: current trends and future perspectives. *Trends in Biotechnology*.

[2] Ganda E. K., Bisinotto R. S., Decter D. H., Bicalho R. C. (2016). Evaluation of an on-farm culture system (Accumast) for fast identification of milk pathogens associated with clinical mastitis in dairy cows. *PLoS One*.

[3] Derakhshani H., Fehr K. B., Sepehri S. (2018). Invited review: microbiota of the bovine udder: contributing factors and potential implications for udder health and mastitis susceptibility. *Journal of Dairy Science*.

[4] Kauf A. C., Vinyard B. T., Bannerman D. D. (2007). Effect of intramammary infusion of bacterial lipopolysaccharide on experimentally induced Staphylococcus aureus intramammary infection. *Research in Veterinary Science*.

[5] Jiang A., Zhang Y., Zhang X. (2020). Morin alleviates LPS-induced mastitis by inhibiting the PI3K/AKT, MAPK, NF-*κ*B and NLRP3 signaling pathway and protecting the integrity of blood-milk barrier. *International Immunopharmacology*.

[6] Song X., Zhang W., Wang T. (2014). Geniposide plays an anti-inflammatory role via regulating TLR4 and downstream signaling pathways in lipopolysaccharide-induced mastitis in mice. *Inflammation*.

[7] Sordillo L. M. (2018). Mammary Gland immunobiology and resistance to mastitis. *The Veterinary Clinics of North America. Food Animal Practice*.

[8] Serou M. J., DeCoster M. A., Bazan N. G. (1999). Interleukin-1 beta activates expression of cyclooxygenase-2 and inducible nitric oxide synthase in primary hippocampal neuronal culture: platelet-activating factor as a preferential mediator of cyclooxygenase-2 expression. *Journal of Neuroscience Research*.

[9] Shi J., Gao W., Shao F. (2017). Pyroptosis: gasdermin-mediated programmed necrotic cell death. *Trends in Biochemical Sciences*.

[10] Lamkanfi M., Dixit V. M. (2014). Mechanisms and functions of inflammasomes. *Cell*.

[11] Bergsbaken T., Fink S. L., Cookson B. T. (2009). Pyroptosis: host cell death and inflammation. *Nature Reviews. Microbiology*.

[12] Aglietti R. A., Estevez A., Gupta A. (2016). GsdmD p30 elicited by caspase-11 during pyroptosis forms pores in membranes. *Proceedings of the National Academy of Sciences of the United States of America*.

[13] Wallach D., Kang T. B., Dillon C. P., Green D. R. (2016). Programmed necrosis in inflammation: toward identification of the effector molecules. *Science*.

[14] Strowig T., Henao-Mejia J., Elinav E., Flavell R. (2012). Inflammasomes in health and disease. *Nature*.

[15] von Moltke J., Ayres J. S., Kofoed E. M., Chavarria-Smith J., Vance R. E. (2013). Recognition of bacteria by inflammasomes. *Annual Review of Immunology*.

[16] Liston A., Masters S. L. (2017). Homeostasis-altering molecular processes as mechanisms of inflammasome activation. *Nature Reviews. Immunology*.

[17] Schroder K., Tschopp J. (2010). The inflammasomes. *Cell*.

[18] Shi J., Zhao Y., Wang K. (2015). Cleavage of GSDMD by inflammatory caspases determines pyroptotic cell death. *Nature*.

[19] Man S. M., Kanneganti T. D. (2015). Regulation of inflammasome activation. *Immunological Reviews*.

[20] Vanaja S. K., Rathinam V. A., Fitzgerald K. A. (2015). Mechanisms of inflammasome activation: recent advances and novel insights. *Trends in Cell Biology*.

[21] van Soest F. J. S., Abbeloos E., McDougall S., Hogeveen H. (2018). Addition of meloxicam to the treatment of bovine clinical mastitis results in a net economic benefit to the dairy farmer. *Journal of Dairy Science*.

[22] Mushtaq S., Shah A. M., Shah A. (2018). Bovine mastitis: an appraisal of its alternative herbal cure. *Microbial Pathogenesis*.

[23] Fitzpatrick C. E., Chapinal N., Petersson-Wolfe C. S. (2013). The effect of meloxicam on pain sensitivity, rumination time, and clinical signs in dairy cows with endotoxin-induced clinical mastitis. *Journal of Dairy Science*.

[24] Zhou Q., Song W., Xiao W. (2017). Dioscin induces demethylation of DAPK-1 and RASSF-1alpha genes via the antioxidant capacity, resulting in apoptosis of bladder cancer T24 cells. *EXCLI journal*.

[25] Yang B., Xu B., Zhao H. (2018). Dioscin protects against coronary heart disease by reducing oxidative stress and inflammation via Sirt1/Nrf2 and p38 MAPK pathways. *Molecular Medicine Reports*.

[26] Qiao Y., Xu L., Tao X. (2018). Protective effects of dioscin against fructose-induced renal damage via adjusting Sirt3-mediated oxidative stress, fibrosis, lipid metabolism and inflammation. *Toxicology Letters*.

[27] Tao X., Wan X., Xu Y. (2014). Dioscin attenuates hepatic ischemia-reperfusion injury in rats through inhibition of oxidative-nitrative stress, inflammation and apoptosis. *Transplantation*.

[28] Zheng L., Han X., Hu Y. (2019). Dioscin ameliorates intestinal ischemia/reperfusion injury via adjusting miR-351-5p/MAPK13-mediated inflammation and apoptosis. *Pharmacological Research*.

[29] Yin W., Liu S., Dong M. (2020). A new NLRP3 inflammasome inhibitor, dioscin, promotes osteogenesis. *Small*.

[30] Ran X., Li Y., Chen G. (2018). Farrerol ameliorates TNBS-induced colonic inflammation by inhibiting ERK1/2, JNK1/2, and NF-kappaB signaling pathway. *International journal of molecular sciences*.

[31] Huang B., Liu J., Ju C. (2017). Licochalcone A prevents the loss of dopaminergic neurons by inhibiting microglial activation in lipopolysaccharide (LPS)-induced Parkinson’s disease models. *International journal of molecular sciences*.

[32] Huang B., Liu J., Meng T. (2018). Polydatin prevents lipopolysaccharide (LPS)-induced Parkinson’s disease via regulation of the AKT/GSK3*β*-Nrf2/NF-*κ*B signaling axis. *Frontiers in Immunology*.

[33] Chung S. D., Lai T. Y., Chien C. T., Yu H. J. (2012). Activating Nrf-2 signaling depresses unilateral ureteral obstruction-evoked mitochondrial stress-related autophagy, apoptosis and pyroptosis in kidney. *PLoS One*.

[34] Ni Y. L., Shen H. T., Su C. H. (2019). Nerolidol suppresses the inflammatory response during lipopolysaccharide-induced acute lung injury via the modulation of antioxidant enzymes and the AMPK/Nrf-2/HO-1 pathway. *Oxidative Medicine and Cellular Longevity*.

[35] Wu S., Xu H., Peng J. (2015). Potent anti-inflammatory effect of dioscin mediated by suppression of TNF-*α*-induced VCAM-1, ICAM-1and EL expression via the NF-*κ*B pathway. *Biochimie*.

[36] Gu B. B., Miao J. F., Zhu Y. M., Deng Y. E., Zou S. X. (2009). Protective effect of retinoid against endotoxin-induced mastitis in rats. *Inflammation Research*.

[37] Kan X., Liu B., Guo W. (2019). Myricetin relieves LPS-induced mastitis by inhibiting inflammatory response and repairing the blood-milk barrier. *Journal of Cellular Physiology*.

[38] Wang X., Feng S., Ding N. (2018). Anti-inflammatory effects of berberine hydrochloride in an LPS-induced murine model of mastitis. *Evidence-based Complementary and Alternative Medicine*.

[39] Yang C., Liu P., Wang S. (2018). Shikonin exerts anti-inflammatory effects in LPS-induced mastitis by inhibiting NF-*κ*B signaling pathway. *Biochemical and Biophysical Research Communications*.

[40] Liu X., Jin X., Yu D., Liu G. (2019). Suppression of NLRP3 and NF-*κ*B signaling pathways by *α*-Cyperone via activating SIRT1 contributes to attenuation of LPS-induced acute lung injury in mice. *International Immunopharmacology*.

[41] Yu S., Liu X., Yu D., Changyong E., Yang J. (2020). Morin protects LPS-induced mastitis via inhibiting NLRP3 inflammasome and NF-*κ*B signaling pathways. *Inflammation*.

[42] Yamamoto Y., Gaynor R. B. (2001). Therapeutic potential of inhibition of the NF-kappaB pathway in the treatment of inflammation and cancer. *The Journal of Clinical Investigation*.

[43] Gross O., Thomas C. J., Guarda G., Tschopp J. (2011). The inflammasome: an integrated view. *Immunological Reviews*.

[44] Bauernfeind F. G., Horvath G., Stutz A. (2009). Cutting edge: NF-kappaB activating pattern recognition and cytokine receptors license NLRP3 inflammasome activation by regulating NLRP3 expression. *Journal of Immunology*.

[45] Abderrazak A., Syrovets T., Couchie D. (2015). NLRP3 inflammasome: from a danger signal sensor to a regulatory node of oxidative stress and inflammatory diseases. *Redox Biology*.

[46] Schneider K. S., Groß C. J., Dreier R. F. (2017). The inflammasome drives GSDMD-independent secondary pyroptosis and IL-1 release in the absence of caspase-1 protease activity. *Cell Reports*.

[47] Chen Y. L., Xu G., Liang X. (2016). Inhibition of hepatic cells pyroptosis attenuates CLP-induced acute liver injury. *American Journal of Translational Research*.

[48] Haldar S., Dru C., Choudhury D. (2015). Inflammation and pyroptosis mediate muscle expansion in an interleukin-1*β* (IL-1*β*)-dependent manner. *The Journal of Biological Chemistry*.

[49] Liu Z., Gan L., Xu Y. (2017). Melatonin alleviates inflammasome-induced pyroptosis through inhibiting NF-kappaB/GSDMD signal in mice adipose tissue. *Journal of Pineal Research*.

[50] Wu S., Zhao F., Zhao J. (2019). Dioscin improves postmenopausal osteoporosis through inducing bone formation and inhibiting apoptosis in ovariectomized rats. *Bioscience Trends*.

[51] Jia Y., Cui R., Wang C. (2020). Metformin protects against intestinal ischemia-reperfusion injury and cell pyroptosis via TXNIP-NLRP3-GSDMD pathway. *Redox Biology*.

[52] de Souza L. F., Barreto F., da Silva E. G. (2007). Regulation of LPS stimulated ROS production in peritoneal macrophages from alloxan-induced diabetic rats: involvement of high glucose and PPARgamma. *Life Sciences*.

[53] Fialkow L., Wang Y., Downey G. P. (2007). Reactive oxygen and nitrogen species as signaling molecules regulating neutrophil function. *Free Radical Biology & Medicine*.

[54] Guo W., Liu J., Sun J. (2020). Butyrate alleviates oxidative stress by regulating NRF2 nuclear accumulation and H3K9/14 acetylation via GPR109A in bovine mammary epithelial cells and mammary glands. *Free Radical Biology & Medicine*.

[55] Dang X., He B., Ning Q. (2020). Alantolactone suppresses inflammation, apoptosis and oxidative stress in cigarette smoke-induced human bronchial epithelial cells through activation of Nrf2/HO-1 and inhibition of the NF-*κ*B pathways. *Respiratory Research*.

[56] Hu Q., Zhang T., Yi L., Zhou X., Mi M. (2018). Dihydromyricetin inhibits NLRP3 inflammasome-dependent pyroptosis by activating the Nrf2 signaling pathway in vascular endothelial cells. *BioFactors*.

[57] Liu Q., Lv H., Wen Z., Ci X., Peng L. (2017). Isoliquiritigenin activates nuclear factor erythroid-2 related factor 2 to suppress the NOD-like receptor protein 3 inflammasome and inhibits the NF-*κ*B pathway in macrophages and in acute lung injury. *Frontiers in Immunology*.

