# Supplementary material for: Dioscin Improves Pyroptosis in LPS-Induced Mice Mastitis by Activating AMPK/Nrf2 and Inhibiting the NF-κB Signaling Pathway
Source: Oxid Med Cell Longev. 2020 Dec 31;2020:8845521. doi: 10.1155/2020/8845521 (PMC7790561; doi:10.1155/2020/8845521)
Supplement: Supplementary Materials — Supplementary Figure S1: the cell cytotoxicity of dioscin on mMECs. (a) Chemical structure of dioscin. (b) MMEC viability was determined by CCK-8 assay. #p < 0.05 and ##p < 0.01 compared to the control group. Supplementary Figure S2: the gene expression of NLRP3 inflammasome and GSDMD. (a–c) The mRNA levels of NLRP3, ASC, and caspase-1. (d) The mRNA levels of caspase-3. (e) The mRNA levels of GSDMD. #p < 0.05 and ##p < 0.01 compared to the control group. ∗p < 0.05 and ∗∗p < 0.01 compared to the LPS+ATP group. [file 8845521.f1.docx]

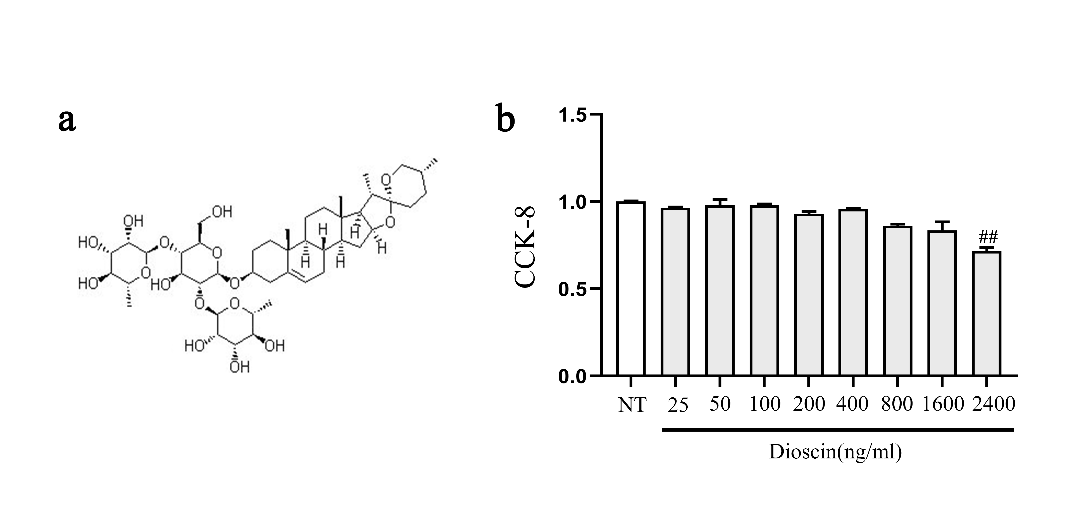


**supplementary** **Figure S1.** The cell cytotoxicity of dioscin on mMECs. (a) Chemical structure of dioscin. (b) MMECs viability were determined by CCK-8 assay. #*p* < 0.05 and ##*p* < 0.01 compared to the control group.

The chemical structure of dioscin is shown in Figure S1 a. In order to detect the toxic effect of dioscin on mMECs, different concentrations of dioscin were used to stimulate mMECs for 24h. The results showed that dioscin had no toxic effect on mMECs when the concentration of dioscin was lower than 2400ng/ml (supplementary Figure S1 b).


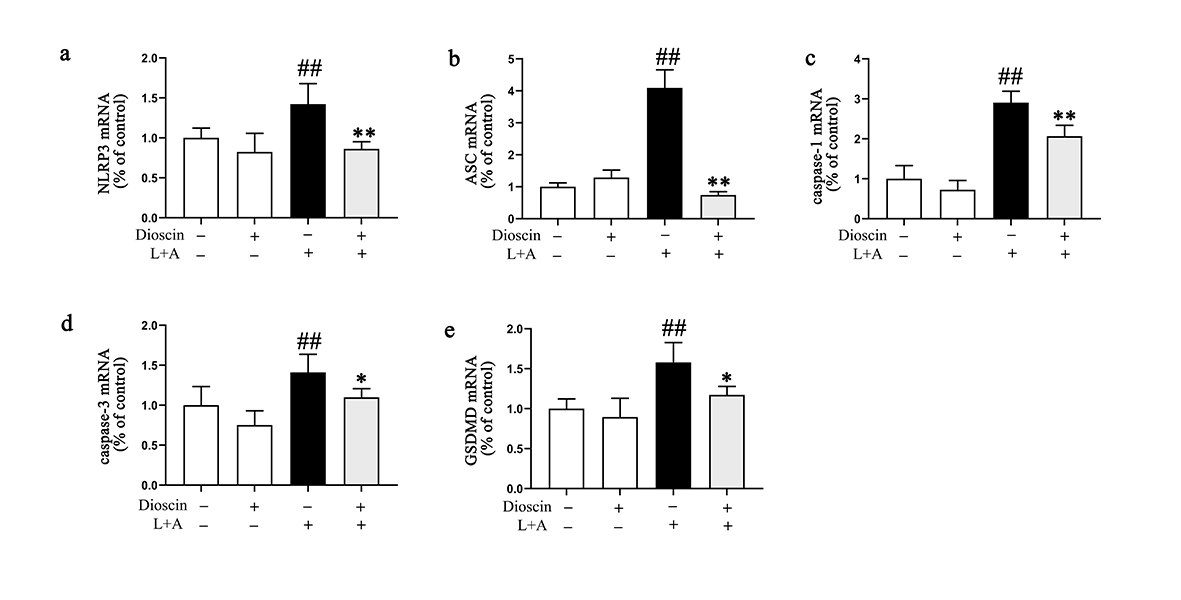
**supplementary Figure S2.** The gene expression of NLRP3 inflammasome and GSDMD. (a-c) The mRNA levels of NLRP3, ASC, caspase-1. (d) The mRNA levels of caspase-3. (e) The mRNA levels of GSDMD. #*p* < 0.05 and ##*p* < 0.01 compared to the control group, **p* < 0.05 and ***p* < 0.01 compared to the LPS+ATP group.

The gene expression of NLRP3, ASC and caspase-1 increased significantly after LPS+ATP stimulation, however dioscin significantly reduced these genes expression induced by LPS+ATP (supplementary Figure S2 a-c). As expected, the gene expression of GSDMD and caspase-3 induced by ATP+LPS was also inhibited by dioscin (supplementary Figure S2 d,c).


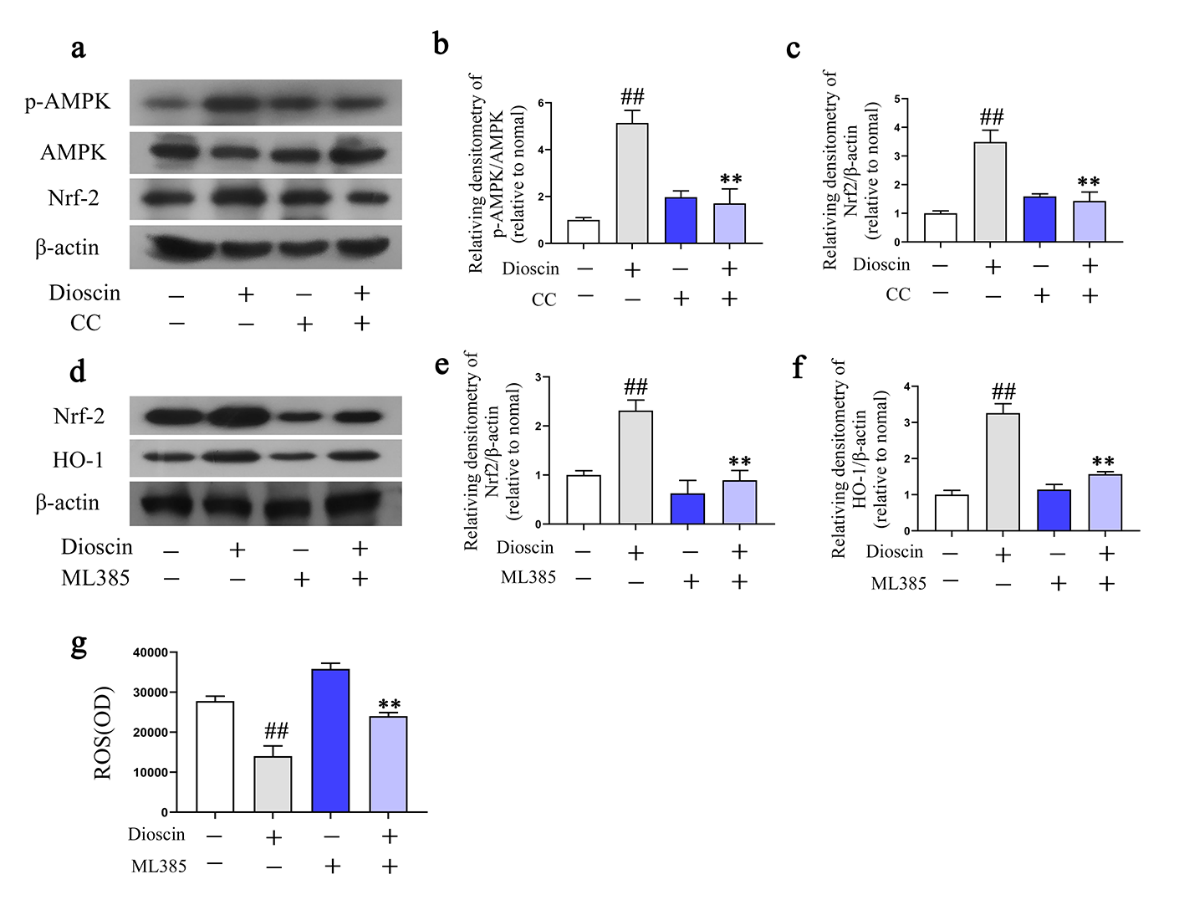


**supplementary Figure S3.** Dioscin activates the p-AMPK/ Nrf2/HO-1 signaling axis in mMECs. The mMECs were treated with CC (1μM) for 2 hours before adding Dioscin(400ng/ml) for 2 hours. (a-c) The protein of p-AMPK, AMPK, Nrf-2 were detected by western blot. The mMECs were treated with CC (1μM) for 4 hours before adding Dioscin(400ng/ml) for 2 hours. (d-f) The protein of Nrf-2 and HO-1 were detected by western blot. (g) The ROS content were determined by ROS detection kits. #p < 0.05 and ##p < 0.01 compared to the control group, *p < 0.05 and **p < 0.01 compared to the CC or ML385 group.

The effect of CC, a inhibitor of AMPK, on the expression of Nrf-2 induced by dioscin was tested. The results showed that inhibition of AMPK phosphorylation significantly inhibited the expression of NRF-2 induced by dioscin. Subsequently, we measured the effect of MK385, a inhibitor of Nrf-2, on HO-1 expression and ROS content, observing that dioscin-induced HO-1 expression and decreasing of ROS content were effectively blocked by treatment with MK385. To sum up, these results indicated that dioscin plays an antioxidant role through AMPK- Nrf-2-ROS signaling axis.
